# Fibrinolytic nanocages dissolve clots in the tumor microenvironment, improving the distribution and therapeutic efficacy of anticancer drugs

**DOI:** 10.1038/s12276-021-00688-7

**Published:** 2021-10-19

**Authors:** Junyoung Seo, Jae Do Yoo, Minseong Kim, Gayong Shim, Yu-Kyoung Oh, Rang-Woon Park, Byungheon Lee, In-San Kim, Soyoun Kim

**Affiliations:** 1 Department of Biochemistry and Cell Biology, Cell and Matrix Research Institute, School of Medicine, Kyungpook National University, Daegu, 41944 Republic of Korea; 2 BK21 Plus KNU Biomedical Convergence Program, Department of Biomedical Science, School of Medicine, Kyungpook National University, 680 Gukchaebosang-ro, Jung-gu, Daegu, 41944 Republic of Korea; 3 School of Systems Biomedical Science, Soongsil University, Seoul, 06978 Republic of Korea; 4 College of Pharmacy, Seoul National University, Seoul, 08826 Republic of Korea; 5 Biomedical Research Institute, Korea Institute of Science and Technology, Seoul, 02792 Republic of Korea; 6 KU-KIST School, Korea University, Seoul, 02841 Republic of Korea

**Keywords:** Drug development, Drug delivery

## Abstract

Fibrin, one of the components of the extracellular matrix (ECM), acts as a transport barrier within the core of tumors by constricting the blood vessels and forming clots, leading to poor intratumoral distribution of anticancer drugs. Our group previously developed a microplasmin-based thrombolytic ferritin nanocage that efficiently targets and dissolves clots without causing systemic fibrinolysis or disrupting hemostatic clots. We hypothesized that the thrombolytic nanocage-mediated degradation of fibrin clots in the tumor ECM can lead to enhanced intratumoral drug delivery, especially for nanosized anticancer drugs. Fibrin clot deposition worsens after surgery and chemotherapy, further hindering drug delivery. Moreover, the risk of venous thromboembolism (VTE) also increases. Here, we used thrombolytic nanocages with multivalent clot-targeting peptides and fibrin degradation enzymes, such as microplasmin, to dissolve fibrin in the tumor microenvironment and named them fibrinolytic nanocages (FNCs). These FNCs target tumor clots specifically and effectively. FNCs efficiently dissolve fibrin clots inside of the tumor vessels, suggesting that they can mitigate the risk of VTE in cancer patients. Coadministration of FNC and doxorubicin led to improved chemotherapeutic activity in a syngeneic mouse melanoma model. Furthermore, the FNCs increased the distribution of Doxil/doxorubicin nanoparticles within mouse tumors. These results suggest that fibrinolytic cotherapy might help improve the therapeutic efficacy of anticancer nanomedicines. Thus, microplasmin-based fibrinolytic nanocages are promising candidates for this strategy due to their hemostatic safety and ability to home in on the tumor.

## Cancer: Improving treatment by dissolving clots

Engineered “nanocages” that deliver clot-busting enzymes to tumors can improve the potency of chemotherapy and potentially reduce the risk of life-threatening complications. Many tumors form capillary-constricting clots composed of the protein fibrin, which impede drug penetration and create the potential for fatal venous thromboembolism. Researchers led by Soyoun Kim of Kyungpook National University, Daegu, South Korea, have devised a promising intervention that uses fibrin-degrading enzymes to clear these clogged vessels. The enzyme is delivered in nanocages composed of the protein ferritin coupled to short peptides that selectively bind fibrin, essentially targeting the treatment to clots. When they administered these nanocages to a mouse model of melanoma, the researchers observed a significantly improved response to chemotherapy and greater tumor penetration. This selective fibrin degradation should also eliminate the conditions that predispose patients to thrombembolism.

## Introduction

Cancer patients exhibit an abnormal plasma clot profile, increased clot formation potential, and a decreased fibrinolytic ability to resolve clots. Patients with cancer are at risk of both tumor progression and venous thromboembolism (VTE) and arterial thrombotic (ATE) complications. The risk of VTE is especially high in those receiving chemotherapy, which is the second leading cause of death in cancer patients^1–3^. Significant fibrin deposition has been observed in many solid tumor biopsies, including those from breast, colon, esophageal, kidney, liver, lung, melanoma, and stomach tumors. The degree of deposition is heterogeneous among tumor types and stages: 20–90% of the tumor area is positively stained for fibrin in all examined tumor biopsies^4^. Thus, the tumor-specific presence of fibrin makes it an attractive target for anticancer therapeutics^5^.

On the other hand, the presence of fibrin in the tumor microenvironment can cause increased interstitial fluid pressure and limit the supply, penetration, and distribution of drugs in tumors. Large amounts of hyaluronic acid, fibrin, collagen, and other extracellular matrix (ECM)-related molecules surrounding tumor cells compress the blood vessels in the core of the tumor, hindering drug delivery^6–8^. Several conventional small molecule chemotherapeutics, such as doxorubicin^9^, paclitaxel^10^, and other clinically relevant compounds^11^, exhibit poor distribution throughout solid tumors. These drugs remain limited to areas immediately surrounding blood vessels and miss large regions of the tumor. Their poor intratumoral distribution may significantly weaken their efficacy, contributing to cancer recurrence and the administration of high doses of drugs that causes adverse effects in patients. The transportation and diffusion of nanoscale materials within the tumor tissue is even more limited since their sizes are orders of magnitude larger than conventional chemotherapeutic compounds^12^.

Treatment with ECM hydrolyzing enzymes, such as collagen^13^ and hyaluronic acid^14^, has been shown to recover vascular characteristics and improve blood supply in certain tumor types; however, the use of these enzymes to improve drug penetration has limitations since collagen and hyaluronic acids are ubiquitously expressed. Recently, Kirtane et al. hypothesized that the presence of fibrin in the tumor ECM contributes to the impaired intratumoral distribution of nanoscale drugs and the use of a fibrinolytic enzyme, such as tissue plasminogen activator (tPA), can overcome the poor distribution from nanocarriers^4^. They showed that administration of tPA increases tumor perfusion and enhances the therapeutic efficacy of anticancer nanomedicines in mouse melanoma and lung cancer models.

We previously developed a thrombolytic nanocage (named CLT-sFt-µP thrombolytic nanocage), which contains multivalent fibrin clot-targeting peptides (CLT: CNAGESSKNC) and multivalent microplasmin (µP) proteins using a small version of the ferritin (sFt) platform^15^. CLT recognizes fibrin–fibronectin complexes in clots, µP efficiently dissolves clots, and their assembly into a nanocage-like structure protects the activated µP from its inhibitors. This activated CLT-sFt-µP thrombolytic nanocage showed a prolonged circulatory lifetime and efficiently lysed the preexisting clots in both arterial and venous thrombosis models with high efficacy^15^. Additionally, when administered systemically, the thrombolytic nanocage reduced the bleeding time compared with tPA, indicating that this nanocage is safer than current tPA therapy, which carries the potential risk of systemic hemorrhage^16^.

Ferritin is a cage-like protein assembly with outer and inner diameters of 12 and 8 nm, respectively. Ferritin has been well studied as a promising platform in cancer therapeutics and has several advantages, such as passive accumulation in tumor sites due to the enhanced permeability and retention (EPR) effect and ligation of functional moieties on its surface without disruption of the cage-like architecture. Additionally, its biostability, biodegradability, and low toxicity/immunogenicity make it a better candidate than synthetic nanosized polymers^17,18^.

We hypothesized that the CLT-sFt-µP nanocage proteins could specifically target fibrin clots in tumor sites, lyse fibrin, and improve the distribution and therapeutic efficacy of anticancer drugs. Our study demonstrated that the CLT-sFt-µP nanocage, named fibrinolytic nanocage (FNC), is a promising tool to resolve fibrin-induced interstitial fluid pressure and improve drug penetration into tumors.

## Materials and methods

### Cell culture

Mouse melanoma B16F10 cells (ATCC, Manassas, VA) and human glioblastoma U87MG cells (ATCC, Manassas, VA) were cultured in high-glucose Dulbecco’s modified Eagle’s medium supplemented with 10% fetal bovine serum (FBS), 100 units/mL penicillin, and 100 μg/mL streptomycin in an incubator at 37 °C in a humidified atmosphere containing 5% CO_2_.

### Protein preparation

The CLT-sFt-µP, sFt-µP, µP, and CLT-µP proteins were expressed in *E. coli* origami2 cells, as described previously^15^. To prepare fluorescently labeled proteins, protein samples were conjugated with Flamma 774 or Flamma 456 (Bioacts, Korea) according to the manufacturer’s protocol to perform in vivo targeting or clot binding analysis, respectively.

### Clot binding analysis

The experimental procedures using human blood samples were performed in compliance with institutional guidelines and were approved by the Institutional Review Board of Kyungpook National University (permission no. KNU 2016-6). Clots were formed by mixing CaCl_2_ (10 mM) and thrombin (0.5 U/ml) with fresh frozen plasma (FFP) (0.2 ml) for 1 h at 37 °C in a microtiter plate. Fluorescently (Flamma 456) labeled proteins (0–3 μM) were layered on top of the clots and incubated for 30 min at 37 °C followed by washing to remove the unbound agent. The fluorescence of the bound proteins was monitored at an excitation wavelength of 488 nm and an emission wavelength of 520 nm with a SpectraMax Gemini EM (Molecular Devices).

### In vitro fibrinolysis assay

Fibrinogen (Cayman, MI, USA) and thrombin (Hematology Tech., VT, USA) were purchased. Fibrinogen (5 mg/ml) in saline was solidified by the addition of thrombin (1 unit/ml) in a 4-mm diameter glass tube for 4 h at 37 °C. The fibrinolytic nanocage (FNC) proteins were preactivated with urokinase (uPA) (20:1, w:w) for 1 h at 37 °C. Different amounts of FNC (4, 20, and 100 µg) were added on top of the fibrin gel, and the height of the remaining gel not yet dissolved by FNC was monitored at certain time points (0, 0.5, 1, 2, 4, 6, 8, 10, 12, or 24 h). The amount of uPA (5 µg) required to activate the largest amount of FNC (100 µg) was independently treated as a control. Additionally, Tris-based saline buffer (TBS; 20 mM Tris-HCl pH 8.0, 150 mM NaCl) was used as a control. The fibrin decay of each sample was analyzed using the exponential decay equation (GraphPad v. 9.1.2). The equation is described as Y = (Y0 − Plateau) × exp^(−K × X)^ + Plateau, where Y0 is the decay value (Y) when time (X) is zero, Plateau is the Y value at time infinity, and K is the rate constant.

We performed a Transwell assay to monitor nanoparticle transportation across the fibrin gel during fibrinolysis. A fibrinogen and thrombin mixture was prepared and placed on the upper chamber of the Transwell plate (6.5 mm diameter, 0.4 μm pore size, Costar, Kennebunk, ME) for 4 h at 37 °C to form the fibrin gel. Preactivated FNC in TBS (0, 4, 20, and 100 µg) and 100 μg of wild-type human ferritin heavy chain (wFTH) were added on top of the fibrin gels. The bottom chamber contained only FNC in TBS. After incubation at r.t. for 0, 0.5, 1, 2, 4, 6, 8, and 10 h, the transported wFTH in the bottom chamber was analyzed by western blot using an antihuman ferritin heavy chain antibody (ab65080, Abcam, Cambridge, MA). Urokinase (5 μg) or TBS buffer lacking FNC was supplied in the upper and bottom chambers as controls.

### Animal assays

All animal experiments with mice were performed in compliance with the guidelines of the Institutional Animal Care and Use Committee of Kyungpook National University (permission No. KNU 2020-0055). Male C57BL/6 N mice (6 weeks old) were purchased from Orient Bio Inc. (Seongnam, Rep of Korea). Animals were bred in a pathogen-free facility, and all efforts were made to minimize animal suffering.

### In vivo tumor targeting and biodistribution of the fibrinolytic nanocage

Freshly harvested U87MG cells (1 × 10^6^ cells/mouse) were subcutaneously injected into the mouse flanks to create the tumor xenograft model. CLT-sFt-μP, CLT-μP, CLT-μP, and μP proteins were labeled with Flamma 774 (Bioacts, Korea). Labeled sFt-μP (3.266 mg/kg), CLT-sFt-μP (3.2 mg/kg), μP (2 mg/kg), and CLT-μP (2.066 mg/kg) were intravenously injected into U87MG tumor-bearing mice (*n* = 3 mice/group) via the tail vein. All proteins were injected in equal amounts (0.2 mol) and modified to yield the same fluorescence (by adding unlabeled proteins) to allow for direct comparisons of tumor-targeting efficiency. In vivo fluorescence images were acquired at different time intervals after protein injection (0, 24, 48, 72, 96, 120 h) using an eXplore Optix system (ART Advanced Research Technologies Inc., Montreal, Canada), with excitation/emission wavelengths of 778/805 nm. The obtained images were processed using eXplore Optix OptiView Software and normalized based on the baseline fluorescence assessed for each mouse prior to protein injection.

### In vivo fibrinolysis at the tumor site by the fibrinolytic nanocage

The mice were subcutaneously injected in the flank with freshly harvested B16F10 cells (1 × 10^5^ cells/mouse) to construct the melanoma tumor allograft model. After 14 days, equal molar amounts of proteins, such as CLT-sFt-µP (15 mg/kg daily for 4 days), sFt-µP (14.69 mg/kg daily for 4 days), and CLT-sFt (7.5 mg/kg daily for 4 days), were intravenously injected into B16F10 tumor-bearing mice (*n* = 3 mice per group). Finally, the mice were sacrificed and perfused through the heart, and the tumor tissues were harvested for examination. The tumor sections were first stained with a rabbit polyclonal fibrin antibody (Ogen, Tampa, FL) and a rat polyclonal CD31 antibody (1:100) to visualize fibrin and blood vessels, respectively, followed by staining with an Alexa Fluor 594-conjugated goat anti-rabbit secondary antibody and an Alexa Fluor 488-conjugated goat anti-rat secondary antibody^19^. The nuclei were stained with 4′,6-diamidino-2-phenylindole (DAPI; 1 μg/ml in PBS). Four randomly assigned regions in each tumor slice (*n* = 3) were observed using a Nuance microscope (PerkinElmer, Waltham, MA) at 200× magnification. The fluorescence intensity of fibrin was analyzed, and fibrin deposition was quantified by fluorescence intensity using inForm (v2.1) software (PerkinElmer, Waltham, MA).

### In vivo antitumor efficacy of the fibrinolytic nanocage (FNC)

An in vivo tumor model was established by subcutaneously inoculating B16F10 tumor cells (1 × 10^6^ cells) into the dorsal flanks of 6-week-old female BALB/c mice. When the tumors were ~100 mm^3^ in size, the tumor-bearing mice were intravenously (i.v.) injected with CLT-sFt-µP (FNC) (15 mg/kg) or CLT-sFt (7.5 mg/kg, equivalent to the number of moles of ferritin in a dose of CLT-sFt-µP) every other day for a total of eight injections. The injected amount of FNC was determined according to the corresponding molar concentration of microplasmin used in previous reports where microplasmin exhibited clot-resolving activity in mouse ischemic stroke models^20,21^. CLT-sFt-µP (FNC) was preactivated using uPA before injection. For cotreatment with doxorubicin (Dox), mice were treated with Dox (1 mg/kg) 30 min after either CLT-sFt-µP (FNC) or CLT-sFt injection. Tumor volumes and body weights were measured every other day, and tumor volume was calculated using the following formula: Volume = (Length × Width × Width)/2. Tumor weights were measured at the end of the experiment.

### Doxil accumulation on tumor site

Mice were subcutaneously injected in the flank with freshly harvested B16F10 cells (1 × 10^6^ cells) to construct the tumor allograft model. CLT-sFt-µP (FNC) was preactivated by adding urokinase (uPA) (20:1, w:w) before injection. The same amount of uPA was used as a cotreatment in the Doxil-only group. The preactivated FNC (10 mg/kg, *n* = 4) proteins were intravenously injected into the tumor-bearing mice twice within a 24 h interval. Doxil (doxorubicin, 2.9 mg/kg) was intravenously injected into the mice 30 min later^22,23^. Saline (pH 7.4, *n* = 3) was intravenously injected into the control group. The mice were sacrificed 24 h later, and the tumors were excised. Tumor tissue was fixed with 4% paraformaldehyde (PFA) and embedded in frozen section compound (3801480, Leica, Wetzlar, Germany) to generated cryo-tissue blocks. The tumor sections were stained with an anti-fibrinogen antibody (ab34269, Abcam, Cambridge, MA, USA) followed by treatment with an Alexa Fluor 488-conjugated anti-rabbit IgG antibody (A-11008, Invitrogen, CA, USA). Nuclei were stained with 4′,6-diamidino-2-phenylindole (DAPI; 62248, Invitrogen, CA, USA). Fibrin and Dox were analyzed by confocal microscopy (K1-Fluo RT, Nanoscope Systems Inc., Daejeon, South Korea) at 400× magnification, and the amounts were quantified by fluorescence.

### Statistical analysis

The statistical significance of the tumor growth results was determined using two-way ANOVA followed by Tukey’s test for multiple comparisons using GraphPad Prism (v. 9.1.2). Other comparisons were performed by using one-way ANOVA followed by Tukey’s test for multiple comparisons for more than three groups or by using Student’s *t*-test for comparisons between two groups. *P* values less than 0.05 were considered to be statistically significant.

## Results

### Design, clot targeting, and fibrin binding activity of the fibrinolytic nanocages

**Fig. 1:**
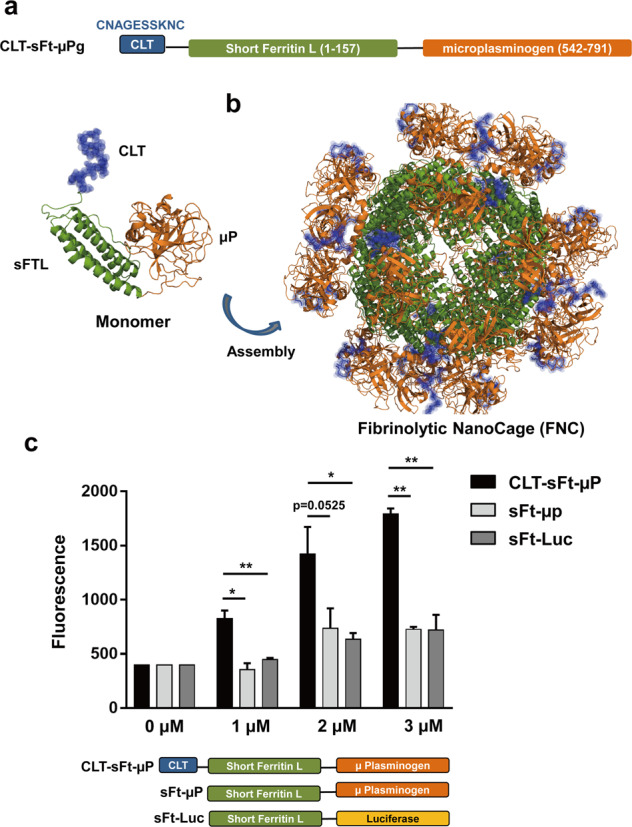
Design of CLT/microplasminogen conjugated ferritin nanocages (CLT-sFt-μP, fibrinolytic nanocages). **a** Schematic diagrams of CLT-sFt-μP showing the clot-targeting peptide (CLT: CNAGESSKNC) ligated to an N-terminal and microplasminogen (μP) conjugated to a C-terminal end of the short version of ferritin (sFt). **b** The 3D model of the fibrinolytic nanocage was drawn using PyMOL v0.99 (CLT peptide, purple; microplasminogen, orange; sFt, green). **c** In vitro fibrin clot binding of CLT-sFt-μP. FITC-labeled proteins (1–3 μM) were added on top of the clots, and the bound proteins were monitored using fluorescence microscopy. The results are presented as the means ± SD (*n* = 3 independent experiments) (one-way ANOVA with Tukey’s test, *P < 0.05, **P < 0.01). Schematic diagrams of the protein constructs described as controls: CLT-lacking nanocage (sFt-μP) and luciferase-conjugated nanocage.

We constructed CLT microplasminogen-carrying ferritin (CLT-sFt-µP) and purified it as previously reported^15^ (Fig. 1a). A structural model of the CLT-sFt-µP assembly was built via computer simulations based on the structure of the wild-type ferritin cage, which contains 24 CLT peptides and 24 microplasminogens on its surface (Fig. 1b). We used the short version of the human ferritin light chain, of which the fifth helix was deleted. Previous reports have shown that the short ferritin (sFt) cage can carry the peptide and protein payloads onto the authentic cage structure without interference with each other’s biological activity and/or avidity^24^. The fibrin binding ability of the CLT-sFt-µP nanocage (fibrinolytic nanocage or FNC) was tested in vitro. To examine whether FNC efficiently targets fibrin clots, CLT-lacking/microplasminogen-carrying ferritin constructs (sFt-µP) and luciferase-conjugated sFt (sFt-Luc) were used as controls. Clots generated from fresh frozen plasma via the addition of CaCl_2_ and thrombin were incubated with fluorescence-labeled proteins. FNC (CLT-sFt-µP) is significantly bound to clots in a dose-dependent manner when compared to controls, indicating that FNC can bind to clots via CLT peptides (Fig. 1c).

### In vitro fibrin digestion by the fibrinolytic nanocage and nanoparticle diffusion into fibrin matrices

**Fig. 2:**
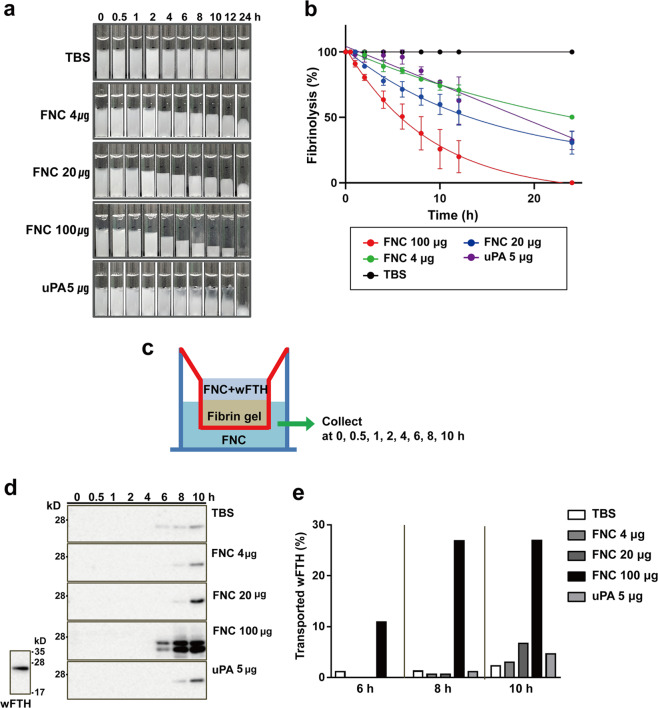
In vitro fibrin dissolution of fibrinolytic nanocages and nanoparticle diffusion. **a** Fibrin dissolved by the fibrinolytic nanocage (FNC) was monitored by measuring the height of the remaining gel after predetermined lengths of time (0, 0.5, 1, 2, 4, 6, 8, 10, 12, or 24 h). Tris-based saline buffer (TBS) and urokinase (uPA) were used as controls. **b** The relative decrease in height of the fibrin gels was plotted. The results are presented as the means ± SD (*n* = 3 independent experiments), and each line was obtained from the exponential decay equation model as described in the Methods. **c** To monitor nanoparticle transport across the fibrin gel upon fibrinolysis, a Transwell assay was performed. FNCs and wild-type ferritin nanocages (wFTHs) were placed on the upper chamber of the Transwell plate. The bottom chamber contained only FNC in TBS. **d** The wFTH transported to the bottom chamber was analyzed by western blot. **e** The relative intensity of the transported wFTH to the applied wFTH was plotted.

To examine fibrinolytic activity, FNC (CLT-sFt-µP) was preactivated by uPA^15^. Our previous study showed that uPA can activate µP in the cage. To assess whether the fibrinolytic nanocage can dissolve fibrin, we laid FNC (4, 20, and 100 µg) onto the fibrin gel and monitored fibrin gel melting at specific time points (Fig. 2a, b). The fibrin gel was melted by the FNC in a dose- and time-dependent manner and remained intact when treated with TBS for up to 24 h (the end of the experiment). To examine a possible nonspecific effect of uPA, 5 µg of uPA (the amount necessary to activate 100 µg of FNC) was independently treated as a control, and the nonspecific effect of uPA was found to be not significant since 5 µg of uPA showed only a moderate effect on fibrin gel digestion. The fibrin decay of each sample was analyzed using the exponential decay equation, and the half-life and decay time constant (Tau) decreased significantly compared to those of uPA alone or the saline controls in a dose-dependent manner (Table 1).

**Table 1:** Fibrin decay parameters using the exponential decay equation.

	FNC (µg)			uPA (µg)	Saline
	100	20	4	5	-
Half-life (h)	6.271	10.24	20.08	19579	*
Tau (h)	9.047	14.78	28.97	28246	*
R^2^	0.9682	0.9599	0.9824	0.9017	1.000

*unstable parameter; Half-life = ln(2)/K; Tau (decay time constant) = 1/K; *R*^2^: coefficient of determination for the goodness of fit of a model, 0 < *R*^2^ < 1.

To study nanoparticle diffusion into fibrin matrices, we used a Transwell assay (Fig. 2c). Previous reports have shown that adding tPA significantly improved nanoparticle movement^4^. Wild-type ferritin heavy chain nanocages (wFTH) were used as model nanoparticles because they can be specifically detected by ferritin heavy chain-binding antibodies by western blot without cross-reactivity with the ferritin light chain, a component of the FNC. We found that the mobility of wFTH was significantly hindered by the fibrin matrix. No wFTH was detected in the receiver chamber for 10 h (Fig. 2d); however, FNC addition seemed to reduce this timeframe. When the amount of FNC added increased at 100 µg, this effect was significantly noticeable (Fig. 2e). In the presence of 5 µg of uPA, wFTH appeared after 10 h of incubation, indicating that the nonspecific effects of uPA were not significant. These experiments show that the fibrin matrix could significantly delay nanoparticle movement and that this effect could be reversed, at least partially, by FNC treatment.

### In vivo tumor-homing ability of the fibrinolytic nanocages

**Fig. 3:**
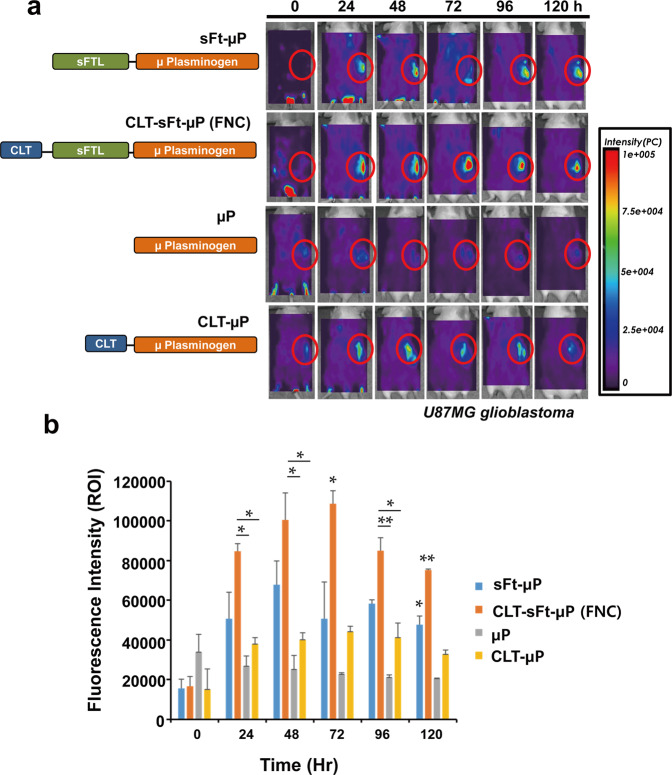
In vivo tumor-homing fibrinolytic nanocages (FNCs) in the U87MG cell xenograft mouse model. **a** Representative images of the biodistribution of CLT-sFt-μP (FNC), CLT-lacking nanocage (sFt-μP), microplasmin protein (μP), and CLT-microplasmin fusion protein (CLT-μP). Mice bearing U87MG tumors were intravenously injected with Flamma 774-labeled proteins followed by in vivo scanning with an eXplore Optix system. **b** The average fluorescence intensity of the tumor region (red circle) was measured. The data represent the means ± SEM. Means of the fluorescence intensities of each group were compared at six incubation times with one-way ANOVA and Tukey’s multiple comparison test (*p < 0.05, **p < 0.01, bars indicate the groups being compared, * or ** without a bar indicates the *p* values among all groups).

Since fluorescence can be hard to detect in the black B16F10 melanoma tumor tissue in vivo, the tumor-homing ability of FNCs was first examined by immunohistochemistry with tumor tissues from B16F10 melanoma-bearing mice that were intravenously injected with FNCs. Immunohistochemical analysis of the tumor tissue showed that the FNCs homed to the tumor sites in both the peripheral and central areas compared to the control (Supplementary Fig. 1a). More FNCs were observed in the peripheral region than in the central region, and quantification of the overall intratumoral FNC supported its tumor-targeting activity (Supplementary Fig. 1b). Since fibrin deposition is widely detected in human cancer tissues and strong fibrin deposition is observed in glioblastoma^25^, a U87MG glioblastoma animal model was generated to further examine tumor targeting in vivo. Mice bearing U87MG glioblastomas were intravenously injected with fluorescently labeled FNC or other control proteins, and whole-body fluorescence images were acquired at different times (Fig. 3a). Microplasmin-conjugated ferritin nanocages without the clot-targeting peptide, sFt-µP, and microplasmin protein with or without clot-targeting peptide, CLT-µP, or µP, respectively, were used as controls. All proteins were injected as preparations containing equal amounts of fluorescence to allow for direct comparisons of tumor-targeting efficiency. Whole-body scans showed that the highest fluorescence signals were detected in FNC-injected animals and that the signals persisted for 120 h. The fluorescence intensity of the regions of interest (red circle) of each group of mice was measured, and the results indicated that the FNCs (CLT-sFt-µP) efficiently targeted the tumor and that CLT conjugation is critical for tumor-homing activity based on comparison with sFt-µP (Fig. 3b). CLT-lacking sFt-µP showed a mild tumor-homing effect, which was attributed to passive delivery via EPR effects. Administration of an equimolar amount of µP proteins did not show specific tumor-homing signals, and CLT-conjugated µP slightly improved targeting efficiency. These results indicate that CLT-conjugated nanoscale FNCs are more effective for tumor homing in animal models.

### In vivo fibrinolysis at the tumor site by the fibrinolytic nanocage

**Fig. 4:**
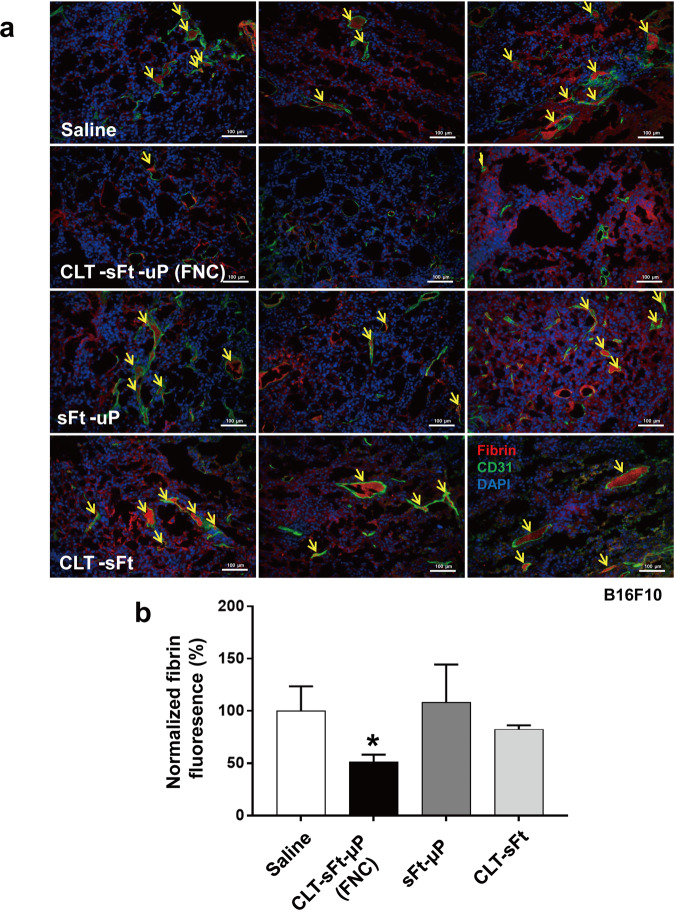
In vivo fibrinolysis at the tumor site by the fibrinolytic nanocage. **a** CLT-sFt-µP (FNC), sFt-µP (CLT-lacking nanocage), and CLT-sFt (microplasmin-lacking nanocage) were intravenously injected into B16F10 tumor-bearing mice. Fibrin deposition (red), blood vessels (green), and nuclei (blue) were visualized in the tumor sections under a Nuance microscope. Yellow arrows indicate fibrin deposition inside of the blood vessels. Scale bars: 100 µm. **b** Fibrin deposition was quantified by fluorescence intensity. The data represent the means ± SEM (*p < 0.05, one-way ANOVA).

To assess whether tumor-homing FNCs dissolve fibrin in the tumor microenvironment, a syngeneic allograft mouse model was constructed by implanting B16F10 mouse melanoma cells into mice, followed by daily intravenous injections of CLT-sFt-µP (15 mg/kg), sFt-µP (14.69 mg/kg), or CLT-sFt (7.5 mg/kg), for a total of four injections. To analyze fibrin deposits in tumor tissue samples, we excised the tumor tissue at the end of the experiments and stained the fibrin and blood vessels. Tumor immunohistochemical analysis showed that the fibrin deposits were much smaller in the FNC-injected mice than in the controls, including the saline, sFt-μP, and CLT-sFt groups (Fig. 4a, b). Specifically, fibrin deposits inside the blood vessels (yellow arrows) were completely cleared when FNC was systemically administered, implying that it efficiently dissolves clots inside tumor blood vessels.

### Enhanced antitumor efficacy of doxorubicin by coadministration with fibrinolytic nanocages

**Fig. 5:**
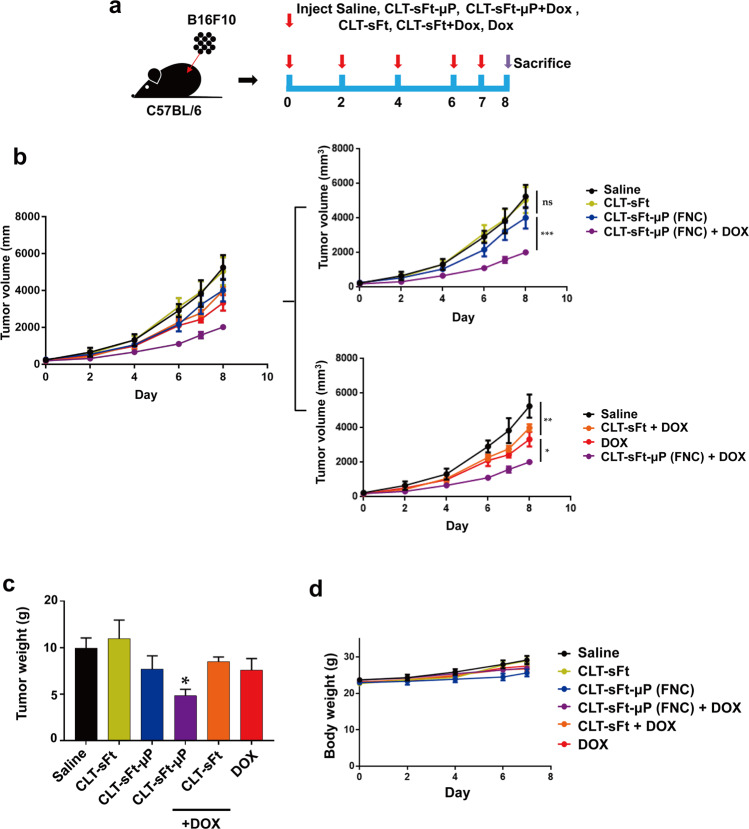
Antitumor therapy with doxorubicin combined with fibrinolytic nanocages. **a** Experimental scheme for antitumor treatments. B16F10 mouse melanoma tumor cells were subcutaneously (s.c.) injected into mice, and treatments were started when the tumor size reached ~100 mm^3^. CLT-sFt-µP (FNC) (15 mg/kg) or CLT-sFt (7.5 mg/kg, equivalent to the number of moles of ferritin in a dose of CLT-sFt-µP) was administered by i.v. injection every other day for a total of eight injections. Doxorubicin (Dox; 1 mg/kg) was injected 30 min after CLT-sFt-µP (FNC) or CLT-sFt injection. **b** Tumor volumes after treatment were plotted into two subgroup graphs to show comparisons between FNC/Dox cotreated mice and FNC alone- (upper) or Dox alone-treated (below) mice. (*p < 0.05, **p < 0.01, ***p < 0.001; two-way ANOVA). **c** The weights of the excised tumors from each group. **d** Body weights. The data in (**c**, **d**) represent the means ± SEM (*p < 0.05; one-way ANOVA).

Because the combination of fibrinolytic nanocages and chemotherapeutic agents may enhance the antitumor efficacy of chemotherapeutic drugs, we examined whether the cotreatment of doxorubicin (Dox) and FNCs had greater therapeutic efficacy than Dox alone. To accomplish this, B16F10 melanoma tumor-bearing mice were administered various treatment combinations: CLT-sFt-µP (FNC) (15 mg/kg) with or without Dox (1 mg/kg) cotreatment, CLT-sFt (7.5 mg/kg) with or without Dox (1 mg/kg) cotreatment, or Dox treatment alone (1 mg/kg). Proteins and Dox were intravenously injected every other day for a total of five injections once the tumors had reached a volume of 100 mm^3^ (Fig. 5a). Only FNC/Dox cotreated mice showed significantly inhibited tumor growth, whereas both Dox or FNC alone showed statistically insignificant tumor growth inhibition compared to saline controls (Fig. 5b). We also observed that mice coinjected with FNC and Dox showed a significant decrease in tumor weight compared to mice injected with either FNC or Dox alone (Fig. 5c). The antitumor effects of Dox were mild and insignificant because only a subtherapeutic dose of Dox was used here to clarify whether FNC enhanced Dox efficacy. The antitumor activity of free Dox was not affected by cotreatment with the microplasmin-unconjugated ferritin control CLT-sFt. CLT-sFt itself had no effect on tumor growth. No change in body weight was detected in any of the injected mice (Fig. 5d). To examine the enhanced antitumor efficacy of doxorubicin coadministered with FNC in other tumor models, we generated MDA MB 231 breast tumor-bearing mice and administered this same combination treatment (Supplementary Fig. 2). Since the tumor growth rate of MDA MB 231 xenograft models is slower than that of the syngeneic melanoma models, we used 14 injections over 30 days (Supplementary Fig. 2a). FNC alone inhibited tumor growth after the tumor size was ~1.5 cm^3^, but the overall difference was not statistically significant compared with the saline control (Supplementary Fig. 2b). Similar to the B16F10 melanoma tumor models, tumor growth and weights were significantly diminished in Dox-treated mice, and cotreatment with FNC further inhibited tumor growth in MDA MB 231 breast cancer model mice (Supplementary Fig. 2b, c). No change in body weights were detected in any of the injected mice (Supplementary Fig. 2d).

### Enhanced penetration of Doxil in the tumor core after coadministration with fibrinolytic nanocages

**Fig. 6:**
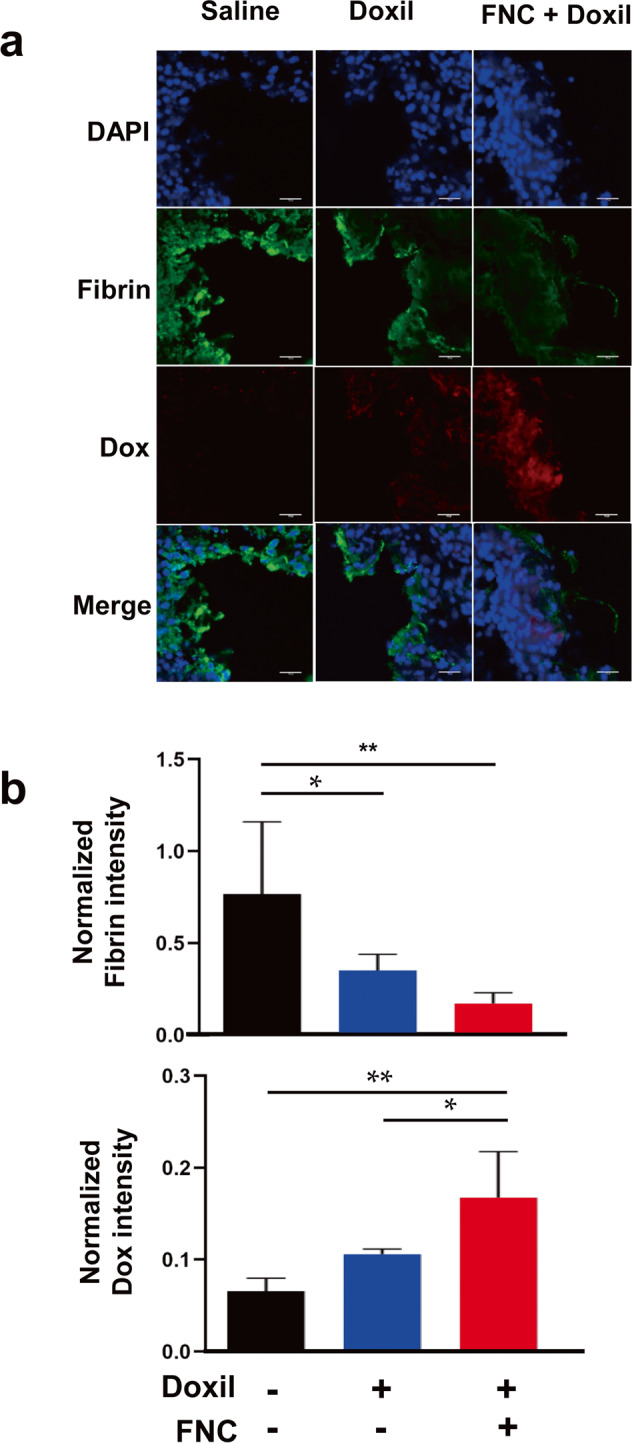
Accumulation of Doxil at the tumor site. **a** Preactivated CLT-sFt-μP (FNC) (10 mg/kg, *n* = 4) proteins and Doxil (doxorubicin, 2.9 mg/kg) were intravenously injected into B16F10 tumor-bearing mice. Fibrin and Dox in the tumor sections were analyzed by confocal microscopy. Scale bars: 30 µm. **b** The amounts of fibrin and Doxil were quantified by fluorescence intensity. The fluorescence intensity of fibrin or Dox was normalized to the fluorescence intensity of DAPI. The data represent the means ± SEM (*p < 0.05, **p < 0.01; one-way ANOVA).

We examined the effect of FNC administration on the intratumoral distribution of nanocarriers using B16F10 melanoma tumor-bearing mice. Doxil was employed in these studies as a model anticancer nanomedicine since doxorubicin fluorescence allows visualization of the drug in tissue sections. Doxil (doxorubicin, 2.9 mg/kg) was coadministered with FNCs (10 mg/kg) or uPA alone (at an equivalent dose for FNC preactivation) as a control. Tumors treated with the combination of FNC and Doxil presented a greater distribution of doxorubicin-associated fluorescence intensity compared to both the control group (Doxil + uPA) and the group treated with saline (Fig. 6a and Supplementary Fig. 3). Fibrin, which had accumulated in the tumor periphery of the control mice, was barely present in the tumor regions of the FNC-treated mice. These results indicate that treatment with FNC resulted in an improved intratumoral distribution of Doxil by dissolving fibrin deposits in the tumor periphery. The total fluorescence intensities of fibrin or Dox in tumors were measured between the Doxil- and Doxil-FNC-treated groups (Fig. 6b), supporting the idea that Doxil distribution throughout the tumor site was improved when the fibrin clots were lysed.

## Discussion

Tumor blood vessels are abnormal and porous due to an imbalance in the pro- and antiangiogenic factors within the tumor tissue^26^. Abnormal vascular architecture and lymphatic vessels in the core of the tumor result in fluid accumulation and cause high interstitial fluid pressure within the tumor site^27^. In addition, a fast-growing tumor cell mass and a highly dense ECM compress blood vessels^8^, further limiting drug delivery due to interstitial fluid pressure and thus lowering the overall efficacy of chemotherapy. Several strategies have been attempted to improve drug delivery within the tumor^28^, including the use of antiangiogenic drugs to normalize blood vessels^29^ and degradation of the ECM in the tumor to improve drug penetration through the tumor matrix. ECM degradation can also alleviate the compression of the collapsed blood vessels, subsequently increasing tumor perfusion^13,14^.

Significant fibrin deposits have been observed within tumors, and their role in cancer development has been suggested^30–33^. During the initial stages of tumor growth, fibrin offers a scaffold for the growth of tumor cells or a cocoon to shield tumor cells from attack by activated lymphocytes. Fibrin also helps angiogenesis, modulating the influx of macrophages and storage of growth factors^34^. Depletion of fibrinogen may decrease the formation of pulmonary metastatic foci owing to the loss of fibrinogen-mediated protection of tumor cells against natural killer cells^32^. The clinical use of anticoagulants and fibrinolytic therapeutics has been attempted to prevent metastasis^35,36^; however, previous studies have implied that only a small portion of tumor sorts respond to anticoagulants and fibrinolytic therapeutics^30^. The main side effect of these treatments is systemic hemorrhage, which is why alternative approaches or tumor-specific therapeutics are necessary for fibrin treatment in tumors. Recently, Kirtane et al. suggested the impact of fibrin deposits in mediating solid stress and limiting drug delivery to tumors. They demonstrated that enzymatic degradation of fibrin using tPA led to increased tumor perfusion and improved the delivery and chemotherapeutic efficacy of paclitaxel^4^.

We studied fibrinolytic nanocages carrying 24-mer peptides of the fibrin degradation enzyme, microplasmin, and 24-mer peptides of a fibrin-targeting peptide. This fibrinolytic nanocage (FNC) showed enhanced anticancer drug activity, which manifested itself through the increased distribution of Doxil/doxorubicin nanoparticles within the tumor. Moreover, the FNCs effectively and specifically targeted tumors through its clot-targeting activity and persisted in the tumor region for 120 h compared to controls, such as CLT-conjugated microplasmin protein or CLT-lacking nanocages. FNCs efficiently dissolved fibrin clots inside of the tumor vessels, suggesting that they can moderate the risk of VTE in cancer patients. It is also worth noting that coadministration of FNC and doxorubicin led to the improved chemotherapeutic activity of doxorubicin in mouse models of melanoma. In agreement with our results, treatment with tPA has been shown to lead to improved chemotherapeutic activity of nanoparticles^4^; however, the use of tPA should be restricted, especially in patients with pathologies associated with intracranial hemorrhage diseases^37,38^. In our previous report, we examined the clot-resolving activity of CLT-sFt-μP (FNC) and its in vivo bleeding side effects in comparison with tPA^15^. Microplasmin (μP) has been known to be well tolerated after developing symptomatic intracerebral hemorrhage because it can be degraded by its natural inhibitor α2-antiplasmin, and systemically administered μP was shown to induce a significantly shorter bleeding time prolongation than tPA^39^. Our previous studies showed that FNCs were more slowly degraded by α2-antiplasmin than free μP, but the in vivo bleeding time was unchanged by the FNCs compared with free μP or saline control, whereas the same dose of tPA administration significantly prolonged the bleeding time. These data suggest that FNCs might be more beneficial than tPA due to its hemostatic safety^15^. Overall, the FNCs described here have significant translational potential to aid in drug penetration by resolving blood clots in the tumor microenvironment. Careful consideration of all these aspects of hemostatic balance is necessary to maximize the therapeutic benefits obtained from this strategy.

## Supplementary information

Supplementary Information
