# Supplementary material for: Fibrinolytic nanocages dissolve clots in the tumor microenvironment, improving the distribution and therapeutic efficacy of anticancer drugs
Source: Exp Mol Med. 2021 Oct 19;53(10):1592–601. doi: 10.1038/s12276-021-00688-7 (PMC8569170; doi:10.1038/s12276-021-00688-7)
Supplement: Supplementary file 1 — Supplementary Information [file 12276_2021_688_MOESM1_ESM.pdf]

Supplementary Information

Fibrinolytic nanocages dissolve clots in the tumor microenvironment, improving the distribution and therapeutic efficacy of anticancer drugs.

Running title: Fibrinolytic nanocages improve drug penetration.

Junyoung Seo[a§], Jae Do Yoo,[a,b§], Minseong Kim[a,b], Gayong Shim[c], Yu-Kyoung Oh[d], Rang-Woon Park[a,b], Byungheon Lee[a,b], In-San Kim[e,f], Soyoun Kim[a,b]*

Supplementary Methods

Intratumoral measurement of the fibrinolytic nanocage (FNC)

Mice were subcutaneously injected in the flank with freshly harvested B16F10 cells (1×10^6 cells) for the construction of the tumor allograft model. The FNC (10 mg/kg, n = 4) proteins were intravenously injected into the tumor-bearing mice. Saline (pH 7.4, n = 3) was intravenously injected into the control group. The mice were sacrificed 24 h later and tumors were excised. Tumor tissue was fixated with 4% paraformaldehyde (PFA) and embedded into frozen section compounds (3801480, Leica, Wetzlar, Germany) to make cryo tissue blocks. The tumor sections were stained with anti-human ferritin light chain antibody (ab109019, Abcam, Cambridge, MA, USA) followed by treatment with Alexa-Flour 488 conjugated anti-rabbit IgG antibody (A-11008, Invitrogen, CA, USA). Nuclei were stained with 4′, 6-diamidino-2-phenylindole (DAPI, 62248, Invitrogen, CA, USA). FNC were analyzed under confocal microscopy (K1-Fluo RT, Nanoscope Systems Inc., Daejeon, South Korea) at ×400 magnification and the amounts were quantified by fluorescence. The fluorescence intensity of FNC was analyzed and its deposition was quantified by fluorescence intensity using the inForm (v2.1) software (Perkinelmer, Waltham, MA).

In vivo anti-tumor efficacy of the fibrinolytic nanocage (FNC).

An *in vivo* tumor model was established by subcutaneously inoculating MDA MB 231 tumor cells (1×10^6 cells) into the dorsal flank of 6-week-old BALB/c female mice. When tumors were approximately 100 mm^3 in size, tumor-bearing mice were intravenously (i.v.) injected with CLT-sFt-μP (FNC) (15 mg/kg) or CLT-sFt (7.5 mg/kg, equivalent to the number of moles of ferritin in a dose of CLT-sFt-μP) every other day for a total of twelve

injections, followed by two more injections with four-day intervals. CLT-sFt-μP (FNC) was pre-activated using uPA before injection. For co-treatment of doxorubicin (Dox), mice were treated with Dox (1 mg/kg) 30 min after either CLT-sFt-μP (FNC) or CLT-sFt injection. Tumor volumes and body weights were measured every other day and tumor volume was calculated using the following formula: Volume = (Length × Width × Width)/2. Tumor weights were measured at the end of the experiments.

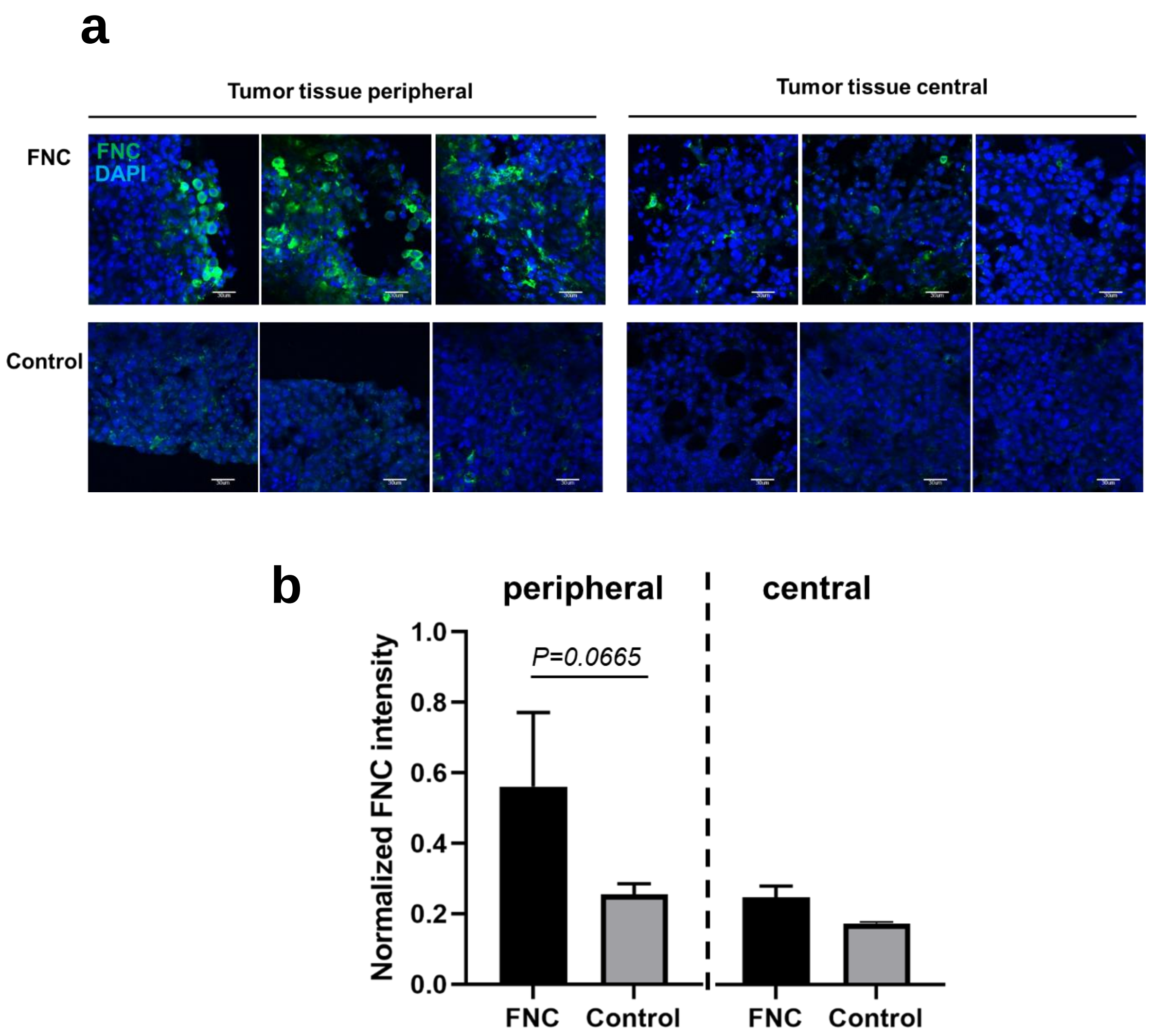

Supplementary Figure 1. Tumor targeting of FNC. (a) Tumor targeted FNC was observed in peripheral or central region of B16F10 tumor tissues from FNC injected mice using anti-human ferritin light chain antibody as described in Method. Scale bars: 30 µm. (b) Amounts of FNC on tumor were quantified by fluorescence intensity of the peripheral and central region. The fluorescence FNC intensity was normalized by fluorescence intensity of DAPI. The data represent means ± SEM (unpaired t-test).

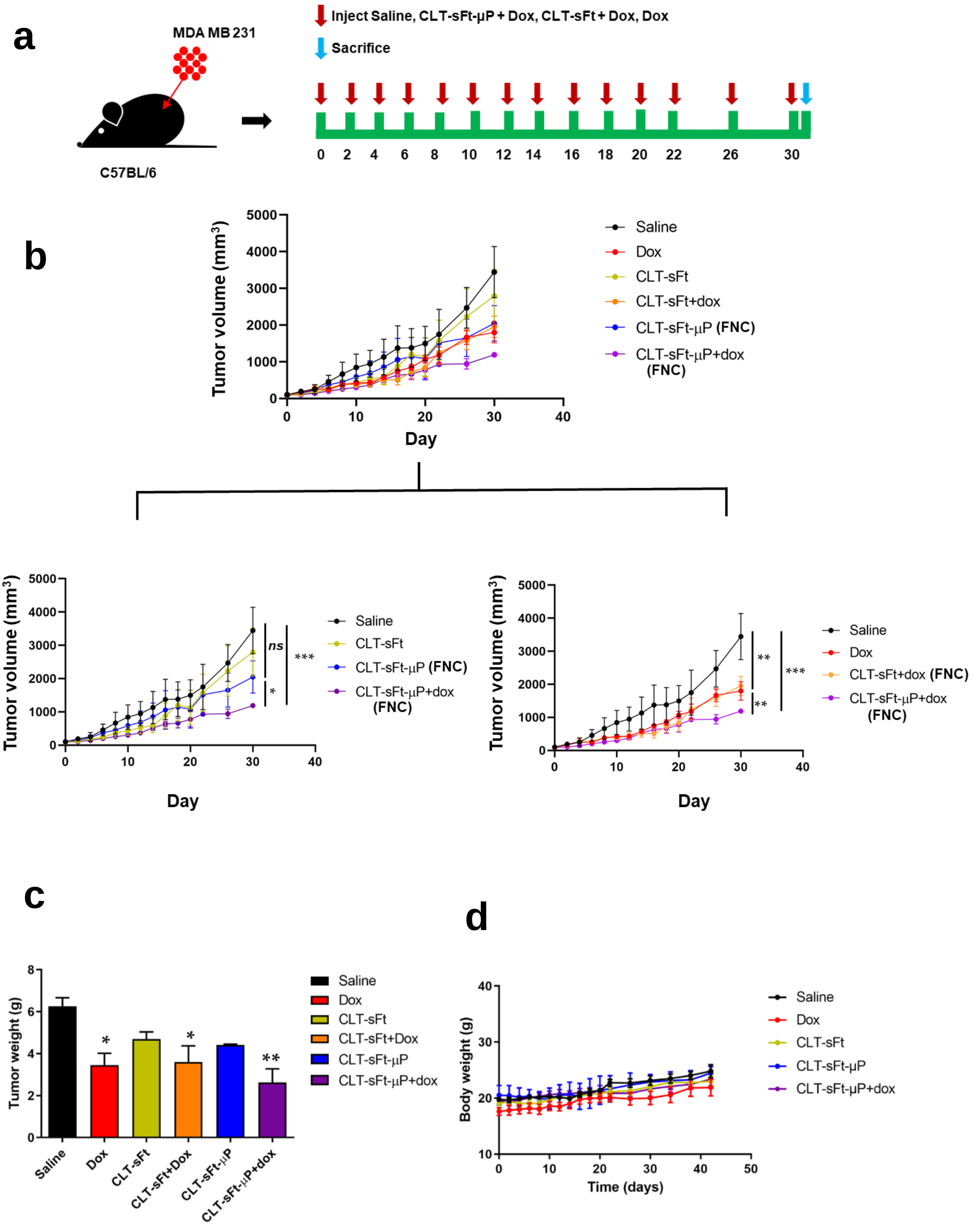

a
MDA MB 231
C57BL/6
Inject Saline, CLT-sFt-μP + Dox, CLT-sFt + Dox, Dox
Sacrifice
0 2 4 6 8 10 12 14 16 18 20 22 26 30
b
Tumor volume (mm3)
Day
Saline
Dox
CLT-sFt
CLT-sFt+dox
CLT-sFt-μP (FNC)
CLT-sFt-μP+dox (FNC)
ns
***
*
CLT-sFt+dox (FNC)
**
c
Tumor weight (g)
Saline
Dox
CLT-sFt
CLT-sFt+Dox
CLT-sFt-μP
CLT-sFt-μP+dox
d
Body weight (g)
Time (days)

Supplementary Figure 2. Anti-tumor therapy of doxorubicin combined with fibrinolytic nanocages with MDA MB 231 breast cancer model.

(a) Experimental scheme for anti-tumor treatments. MDA MB 231 mouse melanoma tumor cells were subcutaneously (s.c.) inoculated into mice, and treatments were started when the tumor size reached approximately 100 mm^3. The CLT-sFt-μP (FNC) or CLT-sFt was administered by fourteen i.v. injections for 30 days. Doxorubicin (Dox, 1 mg/kg) were injected after 30 min of CLT-sFt-μP (FNC) or CLT-sFt injection. (b) Tumor volumes after treatment were plotted in two sub-group graphs to show comparison between FNC/Dox co-treated mice with FNC alone- (left) or Dox alone-treated (right) mice. (*$p<0.05$, ** $p<0.1$, *** $p<0.001$; two-way ANOVA). (c) The weights of excised tumors from each group. (d) Body weights. The data represent means ± SEM (*$p < 0.05$, ** $p<0.1$; one-way ANOVA test) (c,d).

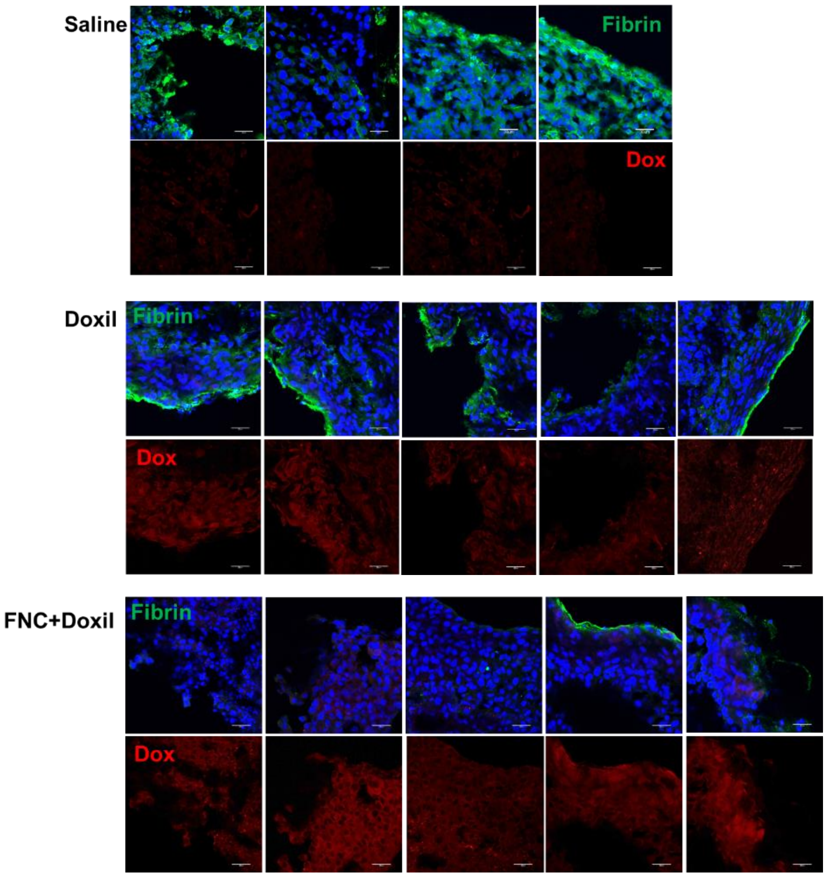

Supplementary Figure 3. Accumulation of Doxil and fibrin deposition on the tumor site

The pre-activated CLT-sFt-μP (FNC) (10 mg/kg, n = 4) proteins and Doxil (Doxorubicin 2.9 mg/kg) were intravenously injected into the B16F10 cells tumor-bearing mice. The images represent fibrin and Dox in the tumor sections under confocal microscopy (K1-Fluo RT, Nanoscope Systems Inc., Daejeon, South Korea) at ×400 magnification. Fibrin was visualized by immunohistochemistry using the anti-fibrinogen antibody as described in Method. Scale bars: 30 μm.
